# Editorial: Innate lymphoid cells: characterization and classification

**DOI:** 10.3389/fimmu.2023.1338463

**Published:** 2023-11-24

**Authors:** Carmelo Luci, Rachel Golub, Carolina C. Jancic

**Affiliations:** ^1^ Université Côte d'Azur, INSERM U1065, Centre Méditerranéen de Médecine Moléculaire, Nice, France; ^2^ Institut Pasteur, Université Paris Cité, INSERM U1223, Paris, France; ^3^ Instituto de Medicina Experimental–Consejo Nacional de Investigaciones Científicas y Técnicas (CONICET)–Academia Nacional de Medicina, Buenos Aires, Argentina; ^4^ Departamento de Microbiología, Parasitología e Inmunología, Facultad de Medicina, Universidad de Buenos Aires, Buenos Aires, Argentina

**Keywords:** innate lymphoid cells, health, disease, classification, development

Innate lymphoid cells (ILCs) are lymphocytes lacking the rearranged antigen receptors and mainly localized at epithelial surfaces, where they maintain tissue homeostasis, and provide a rapid response to pathogen assaults (1). ILCs share similarities with conventional T cells and are divided into 5 subsets based on cell surface markers, transcription factor requirement and ability to produce type 1, type 2 and Th17 cell-associated cytokines: conventional Natural Killer (NK) cells, helper ILC1, ILC2, ILC3 and Lymphocytes Tissue-Inducers (LTi) cells. They are endowed with a plasticity that allows them to modify their phenotype and their functionality to adapt to the microenvironment in which they are located (2). ILCs are considered resident cells in different peripheral tissues but they can also be present in lymph and peripheral blood as it is the case for NK cells (3). The description of the ILCs are complex and discordant since distinctive markers are either not supported in few tissues or across species and after inflammatory conditions. Today, to better understand the origin and classification of ILCs as a whole, and its participation in the immune response, it is necessary to unify criteria and nomenclature after comparing human and mouse recent studies (4).

The goal of this Research Topic was to deepen our knowledge on the origin, classification and activity of ILCs, by focusing on:

- ILCs classification depending on developmental and functional studies.
- ILCs characterization and enlargement to humans.
- How much of ILCs biology is transposable between mice and humans?
- ILCs identity and plasticity during an immune response.
- Diversity in renewal of ILCs subsets during an immune response.
- New functions of specific ILCs subsets (kidney, joints, brain, etc.).

This Research Topic brings together original articles and reviews related to different aspects of ILCs behavior, ranging from their development to their tissue dynamic, including their role in health and diseases. Since the discovery of ILCs, there has been a steady increase in the knowledge about their physiology, which includes their impact on maintaining tissue homeostasis, and their involvement in protection against pathogens and tumors. However, much remains to learn about ILCs, in fields such as ontogenesis, differentiation, migration, among others. Zhang et al. by an integrative analysis of RNA, using scRNA-seq algorithms, identify two gene sets that predominantly differentiate ILCs from CD4^+^ Th subsets, as well as three gene sets that distinguish various immune responses. Authors observe that ILCs and Th subsets are under differential transcriptional regulation. Besides the similarities in effector functions, in ILCs and Th subsets, the underlying regulatory mechanisms exhibit substantial distinctions, supporting the unique roles played by each cell type during immune responses. Additionally, Koprivica et al. focus on the discrepancies in the phenotypic characterization of human and mouse ILC3. The authors analyzed in depth the molecular markers used to identify this population. They discuss the need to unify the definition, isolation, and propagation of ILC3 to increase the possibility of a confluent interpretation of the role of ILC3 in immunity. Calvi et al. provide an overview of the current knowledge about NK cells and helper ILC ontogenesis in humans. They focus on the circulating ILC subsets with killing properties, the unconventional CD56^dim^ NK cells and cytotoxic helper ILCs, and discuss their contribution in both physiological and pathological conditions. Concerning diseases, Roberts et al. summarize the current evidence for the pathological and protective roles of ILCs in cardiovascular disease and its associated risk factor, obesity. Likewise, Wang and Pavert address the steady-state involvement of ILCs in the central nervous system and their participation in major neurological diseases such as ischemic stroke, Alzheimer’s disease, and multiple sclerosis. Concerning the ability of ILC2s to circulate between different organs during inflammation and their potential functions in organs, Mathä et al. review recent findings on ILC2 migration, including their traffic within, into and out of tissues during inflammation, analyzing their roles in mediating multiple type 2 diseases.

A key function of ILCs is their ability to act as a first line of defense during infection, as well as contribute to tissue repair. This is due in part, to their location in the epithelial barriers, skin and mucosa (intestine and lung among others) (5). As a consequence, they may have a pivotal role in the regulation of intestinal homeostasis and in the orchestration of the inflammatory responses. In this Research Topic, and focusing on Inflammatory Bowel disease (IBD), Coman et al. discuss what is currently understood about the roles of helper-like ILC1 in the progression of IBD pathogenesis. As well, authors summarize the published data on helper-like ILC1 plasticity and in their classification in murine and human models. Of note, ILC1s are not the only ILCs involved in gut pathologies, Irie et al. analyze ILC2s, which are mainly associated with parasites immune defense, by studying the global gene expression of ILC2s in health and in colitic conditions using dextran sodium sulfate-induced colitis. Authors reveal the potential roles of type I interferon (T1IFN) in ILC2s during colitis manifestation as T1IFN-stimulated genes were up-regulated in ILC2s. In a similar model of induced colitis, Schroeder et al. reveal the unexpected results that CD90 is not constitutively expressed by functional ILCs in the gut. The authors show that CD90^negative/low^ CD127^+^ ILCs were a potential source of IL-13, IFN-γ and IL-17A at steady state and upon dysbiosis- and dextran sulphate sodium-elicited colitis and could contribute in disease progression. Furthermore, Shi et al. observe that Daikenchuto, one of the most widely used Japanese herbal formulae for various gastrointestinal disorders, restore the reduced colonic ILC3s, mainly RORγthigh-ILC3 in dextran sulphate sodium-elicited colitis model. This herbal formulae attenuates the severity of experimental colitis and maintain the symbiotic microbiota in the colon suggesting that ILC3s play a protective function on colonic inflammation. In accordance with the ability of ILCs to respond to microenvironmental stimuli, Song et al. demonstrate that ILCs are deeply imprinted by their organ of residence, and the conditions specific to healthy or pathological tissues. In the hepatocellular carcinoma microenvironment, authors identified intermediate c-kit^+^ILC2 population, and lin-CD127^-^ NK-like cells that expressed markers of cytotoxicity. Additionally, CD127+CD94^+^ ILC1 were preferentially enriched in inflamed ileum from patients with Crohn’s disease. These analyses provided a baseline for studies focused on tissue-specific ILC-mediated immunity. Notably, there are non-pathologic processes in certain organs, such as in the uterus that require a fine regulation of the immune responses. Interestingly, uterine natural killer cells (uNK) play an important role in promoting successful pregnancy. uNK cells can be divided into three subsets, which may have different roles in pregnancy, and Whettlock et al. establish how uNK frequency and function change dynamically across the healthy reproductive cycle suggesting their implications for the study of subfertility, recurrent miscarriage, and related conditions.

As in many cells, regulation of transcription factors expression needs to be finely controlled to allow ILC development. Conserved non-coding sequences (CNSs) are regulatory cis-acting elements critically controlling gene expression via interaction with various trans-acting factors. The cis-regulatory mechanisms controlling *Rorc* transcription in ILC3s remain unclear. In their study, Chang et al. discover that the deficiency in the conserved noncoding sequence 9 (CNS9) located in *Rorc* gene, selectively decreases RORγt expression in ILC3s. Accordingly, this alters ILC3 gene expression features and promotes cell intrinsic generation of CD4^+^NKp46+ ILC3 subset. This study points CNS9 as an essential cis-regulatory element controlling the lineage stability and plasticity. Contributing to the evidence on the dependence between phenotype and cell localization, Gao et al. uncovered subset-specific differences in the proliferative status between vascular and tissue ILCs within lymphoid and non-lymphoid organs by employing MISTRG humanized mice as an *in vivo* model to study human ILCs. Authors show the proliferative topography of human ILCs, linking cell migration and spatial compartmentalization with cell division. Noticeable, Kabil et al. remark about the plasticity of ILCs, which is controversial due to several confounding caveats that include, among others, the independent large-scale recruitment of new ILC subsets from distal sites and the local, in situ, differentiation of uncommitted resident precursors. To clarify this point, authors detail current methodologies used to study ILC plasticity in mice and review the mechanisms that drive and regulate functional ILC plasticity in response to polarizing signals in their microenvironment.

Collectively, the variety of original papers and reviews presented in this Research Topic have provided a comprehensive overview on ILC physiology and on their behavior in diseases. The insights described here are expected to help understand their role as a key effector in the immune response and their relationship to different pathologies and conditions.

## Author contributions

CL: Writing – review & editing. RG: Writing – review & editing. CJ: Writing – original draft, Writing – review & editing.
